# Measuring Open Porosity of Porous Materials Using THz-TDS and an Index-Matching Medium

**DOI:** 10.3390/s20113120

**Published:** 2020-05-31

**Authors:** Mira Naftaly, Iliya Tikhomirov, Peter Hou, Daniel Markl

**Affiliations:** 1 National Physical Laboratory, Teddington TW11 0LW, UK; mira.naftaly@npl.co.uk (M.N.); iliya.tikhomirov@npl.co.uk (I.T.); 2 Strathclyde Institute of Pharmacy & Biomedical Sciences, University of Strathclyde, Glasgow G1 1XQ, UK; peter.hou@strath.ac.uk; 3 EPSRC Centre for Continuous Manufacturing and Advanced Crystallisation, University of Strathclyde, Glasgow G1 1RD, UK

**Keywords:** terahertz time-domain spectroscopy, porosity, porous media, sensor, quality control

## Abstract

The porosity of porous materials is a critical quality attribute of many products ranging from catalysis and separation technologies to porous paper and pharmaceutical tablets. The open porosity in particular, which reflects the pore space accessible from the surface, is crucial for applications where a fluid needs to access the pores in order to fulfil the functionality of the product. This study presents a methodology that uses terahertz time-domain spectroscopy (THz-TDS) coupled with an index-matching medium to measure the open porosity and analyze scattering losses of powder compacts. The open porosity can be evaluated without the knowledge of the refractive index of the fully dense material. This method is demonstrated for pellets compressed of pharmaceutical-grade lactose powder. Powder was compressed at four different pressures and measured by THz-TDS before and after they were soaked in an index-matching medium, i.e., paraffin. Determining the change in refractive index of the dry and soaked samples enabled the calculation of the open porosity. The results reveal that the open porosity is consistently lower than the total porosity and it decreases with increasing compression pressure. The scattering losses reduce significantly for the soaked samples and the scattering centers (particle and/or pore sizes) are of the order of or somewhat smaller than the terahertz wavelength. This new method facilitates the development of a better understanding of the links between material properties (particles size), pellet properties (open porosity) and performance-related properties, e.g., disintegration and dissolution performance of pharmaceutical tablets.

## 1. Introduction

Many functional materials and products derive characteristic performance-related properties from their microstructure and in particular from their pore structure. Pore structure properties such as internal surface area, pore size, and shape are critical for reaction yields and conversions in catalysis [1], molecular sieves in separation technologies [2], storing greenhouse gases such as CO_2_ in metal organic frameworks (MOFs) [3], as well as in controlling liquid flow in porous paper [4,5] and pharmaceutical tablets [6,7]. The most common descriptor of a pore structure is the porosity, which is defined as the ratio between the volume of the void space to the total volume of the porous material. The porosity can be measured by a number of different techniques spanning across void-sensitive and matrix-sensitive methods. Void sensitive methods utilize a fluid to measure characteristic properties of the pores that are accessible from the surface of the sample, i.e., open pores and open porosity. This type of method includes helium pycnometry [8], mercury porosimetry [9], nitrogen adsorption [10], and thermoporometry [11]. The matrix sensitive methods exploit the interaction of electromagnetic radiation with the porous sample and typically provide a measure of the total porosity (open and closed pores). Some examples of matrix sensitive methods are scanning electron microscopy (SEM) [12], atomic force microscopy [13], focused ion beam SEM [14], confocal laser scanning microscopy [15], nuclear magnetic resonance [16], X-ray computed tomography (CT) [17], and terahertz time-domain spectroscopy (THz-TDS) [18]. It is important to note that most of the matrix-sensitive methods deploy imaging techniques which cannot resolve pores smaller than the optical resolution limit of the instrument. One of the exceptions in the category of the matrix sensitive techniques is THz-TDS, which provides a porosity measure that reflects the total void space including micropores (<2 nm), mesopores (2–50 nm), and macropores (>50 nm) [7]. However, THz-TDS does not provide any information about individual pores or pore size distribution as yielded by imaging-based techniques. 

THz-TDS determines the effective refractive index, $n_{\mathrm{eff}}$, of a porous sample in a non-destructive and contactless manner [19]. This $n_{\mathrm{eff}}$ is directly related to the total porosity and the intrinsic refractive index of the solid matrix. The total porosity can thus be calculated from a THz-TDS measurement by utilizing effective medium theory and knowing the intrinsic refractive index of the solid matrix. This method has been demonstrated to measure the porosity of rocks [20] and pharmaceutical tablets [21,22,23] as well as to analyze the anisotropy of pore structures [18,24]. 

This study demonstrates a new methodology that enables the measurement of the open porosity of a sample using THz-TDS without knowing the intrinsic refractive index of the solid matrix. The open porosity is relevant for many functional materials and products, where a fluid needs to enter the porous medium in order to fulfil its functionality, such as heterogenous catalysts, separation technologies, pharmaceutical tablets and filtering media; or conversely where closed pores are required, as in insulation and barrier media. 

Pharmaceutical grade lactose pellets were compacted at four different pressures to achieve samples with varying porosities. A standard disintegration test was performed to determine the time the pellets require to disintegrate. This performance-related property of a pharmaceutical tablet, i.e., the disintegration time, was correlated with the measured open porosity, indicating that the disintegration performance of the lactose tablet is controlled by its open porosity. 

## 2. Materials and Methods

### 2.1. Sample Preparation

**Figure 1:**
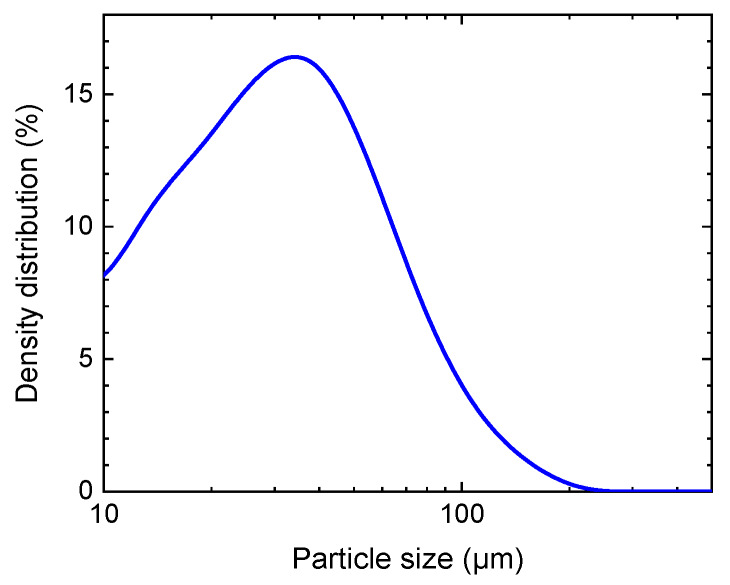
Particle size distribution of lactose powder used in this study.

α-Lactose monohydrate powder (BioReagent grade, Merck) was used to prepare pellets. Pellets were made by compressing 0.200 ± 0.02 g of powder using a hand pellet-maker. Pressure was applied by turning a plunger screw, and was controlled by applying a set torque to the screw-head using a torque wrench. The pressure was calculated from the torque using the screw jack load formula:

(1) $$T=F\times \left[\frac{p+6.28\mu r}{6.28r-\mu p}r\right],\;P=F/A,\;$$

where T is the torque, F is the resulting force on the die, A is the area of the die, and P is the pressure applied to the pellet. The quantities in square brackets are screw parameters: r is the pitch radius, p is the lead of thread, and μ is the friction coefficient. For the pellet-maker employed in this study, the calculated conversion ratio was $P\;\left(N/m^{2}\right)=2\times 10^{6}\;T\;\left(\mathrm{Nm}\right)$. The pellet diameter was 13 mm and their thickness around 1 mm, depending on the pressure. This thickness was optimal for measurement purposes, because thinner pellets were too fragile to handle, while in thicker pellets transmission losses were relatively high. The particle size of the lactose powder was measured using a QICPIC instrument (Sympatec GmbH, Clausthal- Zellerfeld, Germany). The particle size is depicted in Figure 1, and characteristic size properties are as follows: $d_{10}=16.74\pm 0.30$ µm, $d_{50}=45.97\pm 0.16$ µm, and $d_{90}=105.74\pm 1.47$ µm. The $d_{10}$, $d_{50}$, $d_{90}$ are defined as the diameter where 10%, 50%, and 90% of the population lie below the respective value. 

Pellets were prepared using four levels of pressures (four batches labelled as B01–B04) and four pellets per pressure setting (Table 1). The total porosity, $p_{\mathrm{total}}$, of the pellets was calculated using

(2) $$p_{\mathrm{total}}=1-\frac{m}{\pi {\left(\frac{d}{2}\right)}^{2}t\;\varrho_{t}},$$

where d, t, and m are the pellet diameter, thickness, and weight. The true density, $\varrho_{t}$, of lactose was measured using a gas pyncometer with helium (MicroUltrapyc 1200e, Quantachrome instrument, Graz, Austria). The measurement was performed in triplicates and the obtained value was $\varrho_{t}=1.543\pm 0.004$ g/cm^3^, which is in agreement with the literature [25]. This value was used for the porosity calculation in Equation (2).

### 2.2. Terahertz Time-Domain Spectroscopy 

Terahertz optical parameters were measured using THz-TDS. The THz-TDS system was a laboratory-built instrument employing a standard configuration incorporating a Ti:Sapphire femtosecond laser, four off-axis parabolic mirrors, a biased GaAs emitter, and electro-optic detection using a ZnTe crystal, and balanced Si photodiodes. The THz-TDS was frequency-calibrated and had its amplitude linearity verified, as described in [26]. The refractive index and loss coefficient were calculated using a standard formulae [27]. 

### 2.3. Index-Matching Material

**Figure 2:**
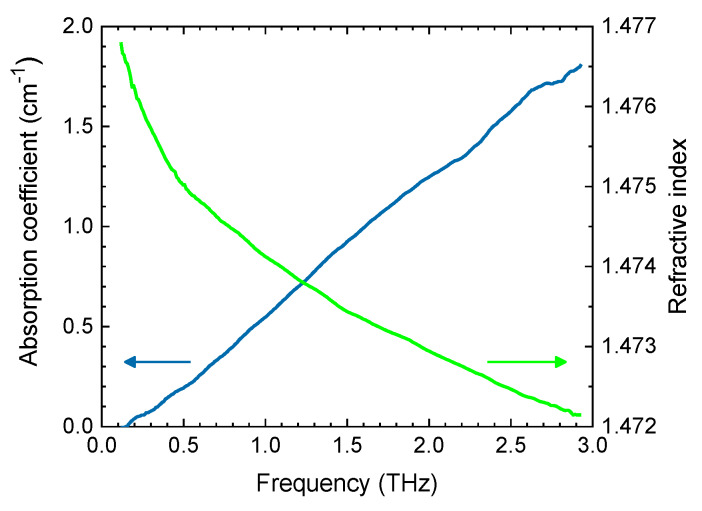
Absorption coefficient and refractive index of paraffin oil.

Paraffin oil was chosen as an index-matching medium because of its high THz transparency and its similar refractive index to that of lactose, as seen in Figure 2. It also has the advantage of a low surface tension of 58 mN/m (compared with 73 mN/m for distilled water), facilitating penetration into microscopic pores. 

### 2.4. Open Porosity Measurement

THz-TDS measurements were first performed on the dry pellets before they were soaked in paraffin for at least 6 h. The pellets loaded with paraffin (henceforth referred to as soaked pellets) were drained on an absorbing tissue before THz-TDS measurements were repeated. Each pellet was measured 10 times; each time it was removed from the sample holder, then replaced to average over positioning errors. This was done to account for both inhomogeneities in the pellets and for slight deviations of tilt off the normal. The data is presented as the mean of all measurements with the uncertainties given as the standard deviation. The THz optics had *F*# = 2, and the THz beam diameter in the plane of the sample was 4 mm. The obtained optical parameters are therefore averaged over the volume of the sample traversed by the beam. The measured refractive index combines contributions from lactose, air in pores, and paraffin in the case of soaked pellets. The measured loss coefficient combines contributions from lactose absorption and from scattering by pores, whereas absorption by paraffin in soaked pellets is negligible. 

### 2.5. Disintegration Testing 

A Copley DTG 2000 Disintegration Tester (Copley Scientific Ltd, Nottingham, UK) was used to measure the disintegration time of tablets in 800 mL of distilled water at 37 °C. Disintegration time was measured individually for five to seven tablets per pressure level. The mean and standard deviation for each pressure level is reported.

## 3. Results

**Figure 3:**
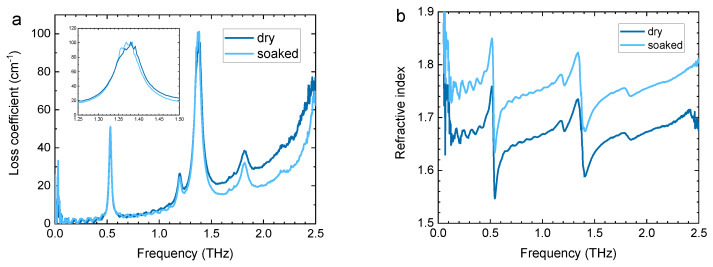
The (**a**) loss coefficient and (**b**) and refractive index of the dry and soaked lactose pellets.

Figure 3 shows the effects of index-matching medium on the loss coefficient and effective refractive index of a lactose pellet. In Figure 3a the loss coefficient combines contributions from absorption and scattering. As expected, the baseline loss decreases with the addition of paraffin, because interface reflection is reduced from 0.027 for lactose-air to 0.006 for lactose-paraffin. The loss difference rises with frequency due to stronger scattering. At the absorption peak, in contrast, shown in the inset, the loss remains constant because the relative contribution of scattering is negligible. However, the effect of reduced scattering is seen in the slight narrowing of the loss peak. The effect of paraffin is also observed in the refractive index (Figure 3b), which increases in the soaked pellets as paraffin replaces air in the open pores.

**Figure 4:**
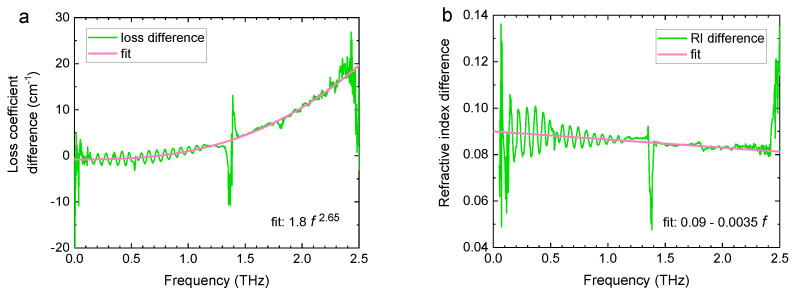
The (**a**) loss coefficient and (**b**) refractive index difference between a lactose pellet when dry and when soaked in paraffin. Solid lines are fits to the data.

A more detailed analysis can be performed by considering the difference between the optical parameters of the dry and soaked lactose pellet, as shown in Figure 4 for a B01 pellet made with a pressure of 3 × 10^7^ N/m^2^. The oscillations are etalon artefacts that do not cancel out due to the mismatch in the refractive indices of the dry and soaked pellet. The loss difference in Figure 4a is seen to increase with frequency. Scattering loss in compressed pellets has been shown to follow a power law [28]. Fitting a power law function to the data in Figure 4b shows that the scattering loss in this case behaves as $1.8f^{2.65}$ with f as the frequency. The power coefficient of 2.65 indicates that the scattering centers (particle and/or pore sizes) are of the order of or somewhat smaller than the wavelength. The refractive index difference in Figure 4b allows a direct estimation of the volume fraction of open pores ($p_{\mathrm{open}}$) using a simple linear model:

(3) $$n_{\mathrm{eff},\mathrm{dry}}=(p_{\mathrm{closed}}+p_{\mathrm{open}})n_{\mathrm{air}}+\left(1-p_{\mathrm{closed}}-p_{\mathrm{open}}\right)n_{\mathrm{lactose}},\;n_{\mathrm{eff},\mathrm{soaked}}=p_{\mathrm{closed}}n_{\mathrm{air}}+p_{\mathrm{open}}n_{\mathrm{paraffin}}+\left(1-p_{\mathrm{closed}}-p_{\mathrm{open}}\right)n_{\mathrm{lactose}},\;\Delta n_{\mathrm{eff}}=p_{\mathrm{open}}(n_{\mathrm{paraffin}}-1),\;p_{\mathrm{open}}=\Delta n_{\mathrm{eff}}/(n_{\mathrm{paraffin}}-1),$$

where $n_{\mathrm{air}}$, $n_{\mathrm{lactose}}$, $n_{\mathrm{paraffin}}$ are the refractive indices of air ($n_{\mathrm{air}}=1$), lactose and paraffin ($n_{\mathrm{paraffin}}=1.475$), $p_{\mathrm{open}}$ and $p_{\mathrm{closed}}$ are the open and closed porosities related to the total porosity as $p_{\mathrm{total}}=p_{\mathrm{open}}+p_{\mathrm{closed}}$. The refractive indices of the dry and soaked pellets are given as $n_{\mathrm{eff},\mathrm{dry}}$ and $n_{\mathrm{eff},\mathrm{soaked}}$. The difference between the refractive index values of the dry and soaked pellets is denoted as $\Delta n_{\mathrm{eff}}$. As an example, this model yields $p_{\mathrm{open}}=\Delta n_{\mathrm{eff}}/(n_{\mathrm{paraffin}}-1)\approx 0.085/\left(1.475-1\right)\approx 0.18$ for B01 pellets.

**Figure 5:**
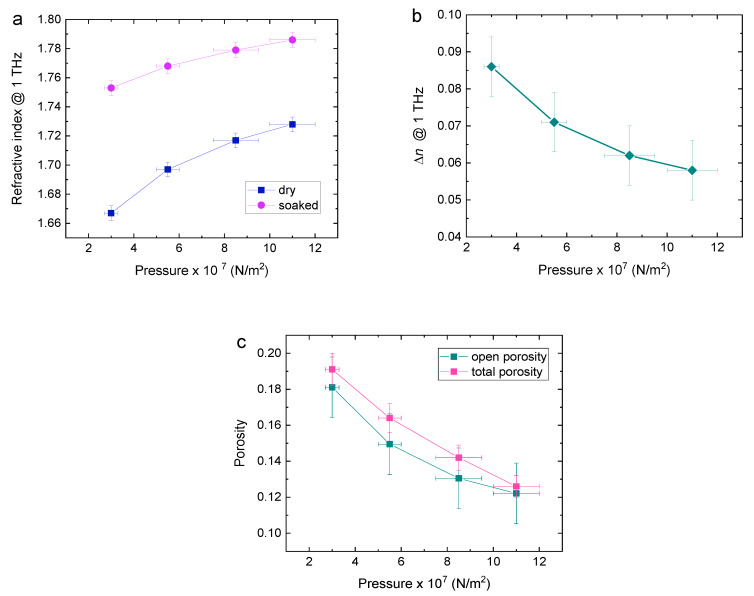
(**a**) Refractive indices of dry and paraffin-soaked pellets @ 1 THz as a function of pressure. The errors in the refractive index are the standard deviation of four pellet samples. (**b**) Refractive index difference @ 1 THz between dry and paraffin-soaked pellets as a function of pressure. (**c**) Total porosity using Equation (2) and open porosity calculated from data in (**b**) and Equation (3), as a function of pressure.

In order to investigate the relationships in more detail, lactose pellets were prepared using four levels of compression pressures, as detailed in Table 1. The total porosity of the resulting pellets is shown in Figure 5c as a function of pressure. It is seen that the reduction in porosity with pressure deviates slightly from linear, showing saturation behavior, as expected. 

The refractive index of dry and paraffin-soaked pellets can then be plotted as a function of pressure (Figure 5). The increase in refractive index in Figure 5a and the difference between refractive indices of dry and soaked pellets in Figure 5b both deviate from linear, as consistent with porosity in Figure 5c, which compares the values of total porosity and the values of open porosity calculated from the data in Figure 5b using Equation (3). It is seen that, as expected, the total porosity consistently exceeds open porosity, except at the highest pressure where the difference lies within the experimental error.

**Figure 6:**
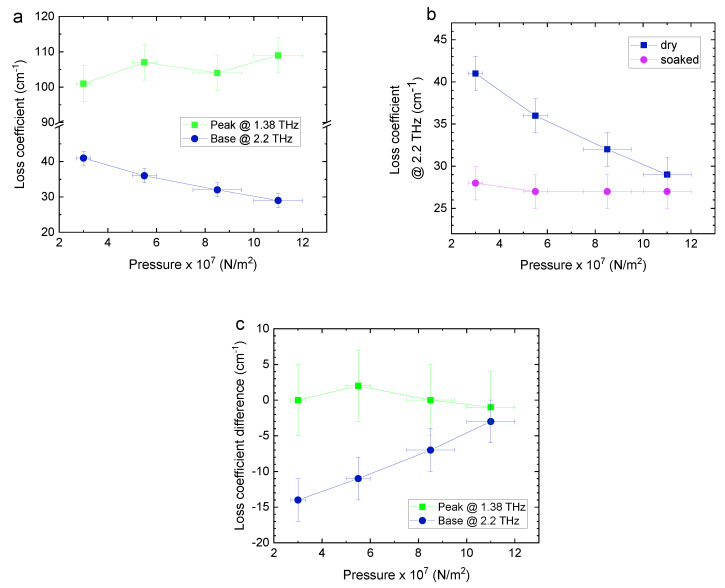
(**a**) Loss coefficients of dry pellets at the absorption peak @ 1.38 THz and away from absorption @ 2.2 THz, as a function of pressure. The errors in the loss are the standard deviation of four pellet samples. (**b**) The loss coefficients of dry and soaked pellets @ 2.2 THz as a function of pressure. (**c**) Loss coefficient difference between dry and paraffin-soaked pellets @ 1.38 THz and @ 2.2 THz as a function of pressure.

Similarly, the loss coefficient, which combines contributions from absorption and scattering, can also be plotted as function of pressure, presented in Figure 6. Figure 6a shows the loss coefficient of dry pellets at the absorption peak @ 1.38 THz and away from absorption @ 2.2 THz. It is seen that at the peak, the loss increases slightly due to more absorbing material in the beam path. In contrast, away from absorption the loss decreases due to reduced scattering as porosity decreases. Focusing on the effects of scattering, Figure 6b presents the loss @ 2.2 THz in dry and paraffin-soaked pellets. This comparison reveals that the use of index-matching medium, where paraffin fills open pores, counteracts scattering due to reduced interface reflection. The loss in soaked pellets becomes constant within measurement uncertainty, except for a small excess at the lowest pressure. The loss coefficient difference between dry and soaked pellets is depicted in Figure 6c. As expected, at the absorption peak @ 1.38 THz there is no measurable difference; whereas away from absorption @ 2.2 THz the difference decreases with pressure as porosity, and therefore scattering, decreases. 

**Figure 7:**
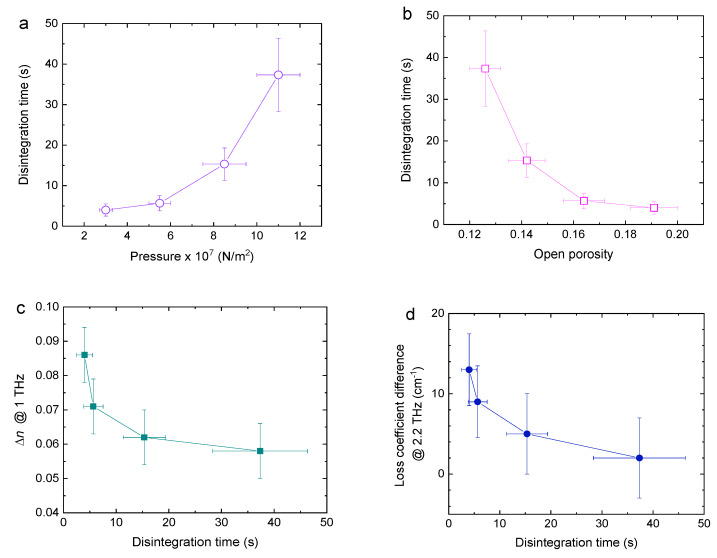
(**a**) Disintegration time of pellets as a function of pressure. (**b**) Disintegration time as a function of open porosity. (**c**) Refractive index difference between dry and paraffin-soaked pellets @ 1 THz as a function of DT. (**d**) Loss coefficient difference between dry and paraffin-soaked pellets @ 2.2 THz as a function of DT. The errors in the refractive index and loss are the standard deviation of four pellet samples.

Disintegration time (DT) measurements is a standard quality control technique in the pharmaceutical industry for evaluating the performance of a tablet. DT is known to largely dependent on the tablet porosity and it is in many cases the rate-determining process [6,7]. The pores in a tablet drive the liquid uptake process, which is necessary to initiate the swelling/dissolution of particles. This swelling/dissolution process eventually causes the break-up of the tablet, i.e., the tablet disintegrates. The liquid uptake process is thus the first and often the rate-determining process and pores accessible from the surface (open pores) will primarily contribute to the rate of this process. DT was measured for lactose pellets produced using the same technique as those used for THz measurements, and the results are shown in Figure 7a. It is seen that DT rises steeply as a function of pressure. This is primarily attributed to the decreasing porosity (Figure 7b) and also to changes in the pore structure [29]. Figure 7c,d respectively plots the refractive index difference and the loss difference as a function of DT. It is seen that both drop steeply at first and then flatten out. However, the loss difference continues to decrease throughout the range of pressures examined, and therefore may offer a better insight and a tool for indirect estimates of DT. 

## 4. Discussion

This study demonstrates the use of THz-TDS coupled with an index-matching medium to analyze the open porosity and scattering losses of compressed lactose powder. The results indicate that the open porosity decreases with increasing compression pressure. This decrease in porosity slows down the disintegration action of the lactose samples as it impedes the uptake of the disintegration medium (in this case water) by the sample. Reducing the rate of the liquid entering the pellet causes a delay in the break-up of the sample and thus prolongs the disintegration time. Understanding and controlling this effect is of paramount importance in the context of the performance of pharmaceutical tablets, where the tablet (or pellet) disintegration is directly linked to the dissolution of the drug substance [6,7,29]. The presented methodology enables a direct measurement of the open porosity and thus facilitates the development of a better understanding of the relationship between material properties (e.g., particle size), tablet properties (porosity) and performance attributes (disintegration and dissolution). 

Previous studies have shown the measurement of the porosity of a sample using THz-TDS through the use of effective medium theory [23] or zero porosity approximation [30]. These approaches can determine the total porosity of a tablet from a THz waveform and the known intrinsic refractive index of the solid material. Typically, this intrinsic refractive index must be estimated using a calibration data set that consists of THz-TDS measurements of pellets compacted at various pressures paired with known total porosity values. This procedure is not required for the proposed method where we use an index-matching medium to determine the open porosity. The open porosity can thus be measured for a single sample without knowing the intrinsic refractive index or the total porosity. However, it is important to note that the proposed method is destructive compared to the non-destructive procedure previously published. 

## 5. Conclusions

Porosity is a critical quality attribute for the majority of pharmaceutical tablets. The open porosity, i.e., the pore space accessible from the surface, is crucial for enabling fluid access and initiating tablet dissolution, which is an essential process in an effective treatment using an orally delivered drug product. This study demonstrated a methodology that uses THz-TDS coupled with an index-matching medium to measure the open porosity and to analyze scattering losses of powder compacts. This method is demonstrated for tablets compressed of pharmaceutical-grade lactose powder using four levels of pressure, and employing paraffin oil as an index-matching medium. We have shown that the open porosity can be evaluated without the knowledge of the refractive index of the fully dense material. Our results reveal that the open porosity is consistently lower than the total porosity. We have also shown that light scattering due to porosity can be separated from absorption losses, making it possible to gain insight into pore size. The scattering centers in the compressed lactose pellets (particle and/or pore sizes) are of the order of or somewhat smaller than the terahertz wavelength. We also show that the disintegration time, a common attribute of pharmaceutical tablets, directly depends on the measured porosity, which highlights the relevance of the demonstrated porosity measurement in the context of pharmaceutical tablets. To the best of our knowledge this is the first time that index-matching fluid has been used to study porosity in pharmaceutical tablets. This new method facilitates the development of a better understanding of the links between material properties (particles size), pellet properties (open porosity) and performance-related properties, e.g., disintegration and dissolution performance of pharmaceutical tablets.

## Figures and Tables

**Table 1:** Pellet thickness and density, applied pressure, and calculated porosity according to Equation (2).

Code	Pressure (N/m^2^) × 10^7^	Thickness (mm) ± 0.01	Density (g/cm^3^) ± 0.02	Porosity
B01	3 ± 0.3	1.21	1.25	0.191 ± 0.009
B02	5.5 ± 0.5	1.17	1.29	0.164 ± 0.007
B03	8.5 ± 0.9	1.14	1.32	0.142 ± 0.006
B04	11 ± 1	1.12	1.35	0.126 ± 0.005
